# Slow fetal growth between first and early second trimester ultrasound scans and risk of small for gestational age (SGA) birth

**DOI:** 10.1371/journal.pone.0184853

**Published:** 2017-09-21

**Authors:** Marija Simic, Olof Stephansson, Gunnar Petersson, Sven Cnattingius, Anna-Karin Wikström

**Affiliations:** 1 Clinical Epidemiology Unit, Department of Medicine Solna, Karolinska University Hospital and Institutet, Stockholm, Sweden; 2 School of Public Health, University of California, Berkeley, California, United States of America; 3 Department of Women’s and Children’s Health, Uppsala University, Uppsala, Sweden; Universitat de Barcelona, SPAIN

## Abstract

**Objectives:**

To investigate the association between fetal growth between first and early second trimester ultrasound scan and the risk of severe small for gestational age (SGA) birth.

**Methods:**

This cohort study included 69 550 singleton pregnancies with first trimester dating and an early second trimester growth scan in Stockholm and Gotland Counties, Sweden between 2008 and 2014. Exposure was difference in biparietal diameter growth between observed and expected at the second trimester scan, calculated by z-scores. Risk of birth of a severe SGA infant (birth weight for gestational age by fetal sex less than the 3^rd^ centile) was calculated using multivariable logistic regression analysis and presented as adjusted odds ratio (aOR).

**Results:**

Parietal growth less than 2.5 percentile between first and second trimester ultrasound examination was associated with elevated risk of being born severe SGA. (aOR 1.67; 95% Confidence Interval 1.28–2.18). The risks of preterm severe SGA (birth before 37 weeks) and term severe SGA (birth 37 weeks or later) were at similar levels, and risk of severe SGA were also elevated in the absence of preeclampsia, hypertensive diseases or gestational diabetes.

**Conclusions:**

Fetuses with slow growth of biparietal diameter at ultrasound examination in early second trimester exhibit increased risk of being born SGA independent of gestational age at birth and presence of maternal hypertensive diseases or diabetes mellitus.

## Introduction

Impaired fetal growth is a major pregnancy complication and determinant of perinatal morbidity and mortality. [1, 2] Increasing evidence suggests that intrauterine growth restriction (IUGR) may have origins in early pregnancy. [3–8] Early-onset IUGR has a strong association with adverse neonatal outcomes, while late-onset IUGR presents with subtle biophysical abnormalities and is therefore more difficult to diagnose. [9–11]

It is biologically plausible that early discrepancy in fetal biometry is a result of placental dysfunction, with impaired fetal growth, resulting in IUGR. Early recognition of fetuses at risk of IUGR enables more appropriate surveillance and management, which has been shown to reduce risks of adverse fetal outcomes. [12, 13] Previous studies have shown that discrepancy in early fetal growth, based on ultrasound scan, is associated with adverse outcomes such as IUGR. [8, 14, 15]However, in these studies, the discrepancy between expected and estimated fetal size was based on comparing information on gestational age based on last menstrual period (LMP) with ultrasound scans. To study early fetal growth restriction, it is preferable to use a more accurate method such as repeated ultrasound measurements, with estimation of change over specified time intervals. [16] In previous research on discrepancy in biparietal diameter (BPD) growth between the first and second trimester ultrasound scans, where an association with small-for-gestational-age (SGA) birth was observed, the focus was on overall risk of SGA, and risks of preterm or term SGA were not studied. [17–21]

We conducted this study to evaluate whether slow growth between the first trimester and the early second trimester based on ultrasound measurements of BPD is associated with increased risks of overall SGA birth, preterm and term SGA birth.

## Materials and methods

### Study design and settings

Data from the population-based Stockholm-Gotland obstetric database was used for the study. The database obtains information from all antenatal, ultrasound, delivery, and postnatal care units in the counties of Stockholm and Gotland, Sweden. From the first antenatal visit, midwives record information about maternal reproductive history, smoking habits, height, weight, and state of health. Biometric measurements, obtained at the ultrasound examinations were collected from all ultrasound units in the region, and information about infant birth weight, maternal diabetes, hypertension and preeclampsia was obtained from standard delivery charts and diagnoses at discharge from the delivery hospital.

### Maternal history and characteristics

The study population included all singleton deliveries in Stockholm and Gotland between January 1st, 2008 and October 22nd, 2014 (n = 167 695). Data on maternal, delivery and infant characteristics was available in of all deliveries. In 72 309 (43%) the pregnancies, biometric measurements were performed both in the first and early second trimesters. We excluded 2 495 pregnancies with infant malformations (3.4%), 198 stillbirths (0.2%) and 66 pregnancies with missing information on infant birth weight. First trimester (12–14 gestational weeks) ultrasound scan is recommended to pregnant mothers older than 35 years to examine the risk for chromosomal disorders (combined ultrasound and biochemistry [CUB] examination). The examination is also available to mothers younger than 35 years on request. All women are offered an early second trimester (18–20 gestational weeks) ultrasound scan in order to calculate the expected date of delivery and to detect multiple pregnancies or fetal malformations. The ultrasound examinations are free of charge and in 2014, 57% of all mothers in Stockholm region, underwent first trimester scans, while 98% underwent early second trimester scans. [22]

The exposure was slow fetal growth defined as the difference between observed and expected BPD at second trimester ultrasound scan. We related the estimated fetal size to the expected size on the basis of the first trimester scan. At both first and second trimester ultrasound examinations, gestational age was estimated by fetal BPD according to the formula by Selbing (gestational age = 58.65 + 1.07*bpd + 0.0138*((bpd) **2). [23] Expected BPD at second trimester scan was calculated by adding number of days between the two examinations to the estimated gestational age at first trimester scan and extrapolated from the formula. The difference between observed and expected BPD was expressed in z scores.

The ultrasound examinations were performed by specially trained midwives according to a standardized protocol. The Swedish Association for Obstetrics and Gynecology (SFOG) recommends that the fetal BPD should be measured from the outer edge of the proximal parietal bone to the inner edge of the distal parietal bone at the level of thalami and septum pelucidi. [24]

### Outcome measures

The primary outcome measure was severe SGA birth, defined as a birth weight less than the 3^rd^ percentile according the sex specific national reference curves for gestational age. [25] The secondary outcome measures were preterm severe SGA birth (infant born before 37 weeks of gestation and SGA) and severe SGA birth at term (after 37 weeks of gestation). Moderate SGA was defined as birth weight < 10^th^ and ≥ 3^rd^ percentiles.

Maternal and pregnancy characteristics, categorized as in Table 1, were maternal age at delivery, parity, early pregnancy body mass index (BMI), maternal height, smoking in early pregnancy, in vitro fertilization (IVF), iatrogenic preterm deliveries, pre-gestational diabetes and hypertension. Pre-pregnancy diabetes mellitus was defined by diagnosis at discharge from the delivery hospital provided in the International Classification of Diseases, tenth revision (ICD-10 codes). Hypertension before pregnancy was defined as treatment with antihypertensive medication at the first antenatal visit or corresponding ICD-10 diagnosis. Hypertension during pregnancy was defined as a blood pressure ≥ 140/90 during pregnancy at two subsequent occasions with at least 6 hours a part. Preeclampsia was defined as new onset of hypertension and proteinuria (+2 or more or +1 measured twice in subsequent samples at least 6 hours apart) after 20 gestational weeks or corresponding ICD-10 diagnosis (O14, O15). ICD codes were provided by the responsible doctor at discharge from the hospital after delivery, while information on blood pressures, proteinuria and medication was provided by midwives at antenatal care or at hospital before delivery.

**Table 1:** Maternal and infant characteristics of the study population according to birth weight.

	Standardized infant birth weight ^b^ (Total N = 69 550)					
Maternal /pregnancy characteristics	> 97^th^ (N = 1 897)	97^th^- >90^th^ (N = 4 830)	90^th^-10^th^ (N = 56 874)	3^rd^—<10^th^ (N = 4 502)	< 3^rd^ (N = 1 447)	p-value ^c^
	n (%)	n (%)	n (%)	n (%)	n (%)	
**Fetal growth** ^a^ **(centile)**						<.0001
> 90^th^	206 (10.8)	514 (10.6)	5 823 (10.2)	426 (9.4)	113 (7.8)	
90^th^ -10^th^	1 485 (78.3)	3 790 (78.5)	45 477 (80.0)	3 599 (80.0)	1 167 (80.6)	
2.5^th^ -10^th^	157 (8.3)	422 (8.8)	4 345 (7.6)	368 (8.2)	115 (7.9)	
< 2.5^th^	49 (2.6)	104 (2.1)	1 229 (2.2)	109 (2.4)	52 (3.4)	
**Age (years)**						<.0001
≤ 24	35 (1.6)	90 (1.9)	1 560 (2.7)	137 (3.1)	57 (3.9)	
25–29	193 (8.7)	518 (10.7)	7 964 (14.0)	690 (15.3)	217 (15.1)	
30–34	734 (33.1)	1 737 (35.9)	21 936 (38.6)	1 769 (39.3)	521 (36.1)	
≥35	1 259 (56.7)	2 485 (51.4)	25 413 (44.7)	1 906 (42.3)	650 (44.9)	
Missing	0	1	1	0	2	
**BMI in early pregnancy (kg/m2)**						<.0001
≤ 18.4	7 (0.4)	48 (1.1)	1 286 (2.4)	182 (4.2)	57 (4.1)	
18.5–24.9	854 (47.1)	2 776 (60.5)	39 381 (72.4)	3 147 (73.2)	1 006 (72.7)	
25–29.9	579 (31.9)	1 248 (27.2)	10 407 (19.1)	732 (17.0)	229 (16.5)	
30–34.9	262 (14.4)	390 (8.5)	2 537 (4.6)	186 (4.3)	58 (4.2)	
≥ 35	114 (6.7)	123 (2.7)	797 (1.5)	56 (1.3)	34 (2.5)	
Missing	81	245	2 466	199	63	
**Height (cm)**						<.0001
130–154	33 (1.7)	59 (1.2)	1 292 (2.3)	214 (4.8)	78 (5.4)	
155–159	90 (4.8)	254 (5.3)	4 751 (8.4)	622 (13.9)	245 (17.1)	
160–169	817 (43.2)	2 243 (46.7)	30 042 (53.2)	2 565 (57.4)	775 (54.1)	
170–200	950 (50.3)	2 240 (46.7)	20 410 (36.1)	1 069 (23.9)	335 (23.4)	
Missing	7	34	379	32	14	
**Nulliparous**	1 474 (77.7)	3 605 (74.6)	31 919 (56.1)	2 828 (62.8)	965 (66.7)	<.0001
**Daily smoker**	30 (1.6)	90 (1.8)	1 353 (2.4)	193 (4.3)	81 (5.8)	<.0001
Missing	11	70	592	41	16	
**In Vitro Fertilization**	119 (6.3)	284 (5.8)	3 510 (6.1)	381 (8.4)	132 (9.1)	<.0001
**Pre-pregnancy Hypertension** ^d^	20 (1.1)	45 (0.9)	481 (0.8)	64 (1.4)	32 (2.2)	<.0001
**Diabetes mellitus** ^e^	41 (2.1)	28 (0.6)	67 (0.1)	5 (0.1)	4 (0.3)	<.0001
**Gestational diabetes mellitus**	144 (7.6)	89 (1.8)	322 (0.5)	19 (0.4)	10 (0.7)	<.0001
**Preterm birth**	75 (2.9)	106 (2.2)	1 876 (3.3)	261 (5.8)	248 (9.7)	<.0001
Missing	8	14	193	27	11	
**Iatrogenic preterm birth** ^f^	32 (1.7)	36 (1.6)	652 (1.1)	156 (3.5)	210 (14.5)	<.0001
**Female sex**	949 (50.1)	2 401 (49.7)	27 736 (48.7)	2 230 (49.5)	736 (50.8)	<.0001
**Preeclampsia/gestational hypertension**	101 (5.3)	179 (3.7)	2 099 (3.7)	356 (7.9)	267 (8.9)	<.0001

^a^ Fetal growth between observed and estimated gestational age at second trimester ultrasound.

^b^ Birth weight according to gestational age and fetal gender.

^c^ p–value linear function.

^d^ Pre-pregnancy hypertensive disease.

^e^ Pre-pregnancy diabetes mellitus.

^f^ Iatrogenic preterm birth including births by C-section and/or induction before 37 weeks of gestation.

### Statistical analyses

We investigated the association between slow fetal growth at early second trimester ultrasound scan and risk for severe SGA birth. A Z-score was calculated for each fetus by subtracting observed BPD and mean of expected BPD and dividing by the standard deviation (SD) of the BPD for this group: Z-score = (observed BPD − expected mean BPD) / (group BPD SD). Slow growth was defined as a BPD measurement at early second trimester less than the 2.5^th^ centile for gestational age.

Unconditional logistic regression analysis was used to calculate crude and adjusted odds ratios (ORs) with 95% confidence intervals (CIs). Adjustments were made for maternal age, BMI, parity, smoking, IVF and maternal pre-pregnancy diabetes and hypertension. Infants were categorized into five groups based on centiles according to birth weight for gestational age and sex: > 97^th^ centile, 97^th^ to > 90^th^ centile, 90^th^ to 10^th^ centile, 3^rd^ to < 10^th^ centile and < 3^rd^ centile. The associations between severe SGA (< 3^rd^ percentiles) and slow BPD growth at early second trimester scan, were investigated using class variables: > 90^th^ centile, 90^th^ to 10^th^ centile (reference), 2.5 ^th^ to < 10^th^ centile and < 2.5^rd^ centile. Mothers with preeclampsia, gestational hypertension or diabetes mellitus, where excluded in the restricted analyses. The analysis of risk of preterm severe SGA was performed in the cohort of all pregnancies. The risk of being born severe SGA at term (≥ 37 weeks) was estimated in the group of term (≥ 37 weeks) pregnancies.

The statistical software package SAS 9.4 (version 6.1; SAS, Cary, NC, USA) was used for analysis.

The regional ethical committee of Karolinska Institutet, Stockholm, Sweden approved the study protocol.

The regional ethical review board of Karolinska Institutet, Stockholm, Sweden approved the study protocol. No written informed consent for participation in the study was obtained from participants since data was depersonalized prior to the analysis. This study was supported by grant from the Swedish research council (Project No. 2013–2429, 2014–3561)

## Results

In total, 69 550 pregnancies were eligible for the study. Based on the information on biometric measurements, 6 950 (10%) of the fetuses had BPD growth less than 2.5^th^ centile at the second trimester ultrasonic scans. In 55 518 (79.8%) of the fetuses, the growth was between 10^th^ and 90^th^ centile, while 10.2% of fetuses had a BPD growth larger than expected (> 90 ^th^ centile).

The characteristics of the study population according to birth weight for gestational age are presented in Table 1. In total, 1 447 infants (2.1%) were severe SGA (< 3^rd^ percentile) at birth. Compared to infants with appropriate birth weight for gestational age (between 90^th^ and 10^th^ percentile), severe SGA infants were more likely slow growing during the early pregnancy. Compared to women delivering infants with normal birth weight for gestational age, mothers delivering severe SGA infants were more likely to be younger (24 years old and younger), lean (BMI ≤ 18.4), cigarette smokers, pregnant after IVF, to deliver preterm, and to have hypertension or preeclampsia.

Compared with the reference group (fetal growth 10^th^ -90 ^th^ centile), the risk of being born severe SGA (<3^rd^ percentile) increased by 70% in infants who had a slow early pregnancy fetal growth (Table 2). In contrast, fetuses with an accelerated fetal growth (< 90^th^ centile) had a lower risk of severe SGA than the reference group. These risks were also present in the analysis restricted to pregnancies without preeclampsia, gestational hypertension or diabetes mellitus (Table 2, restricted model). The fetal growth below 2.5 ^th^ centile did not significantly influence risk of intermediate SGA (3^rd^—<10^th^ percentiles) (S1 Table)

**Table 2:** Risk for severe small for gestational age (SGA) in relation to the fetal growth at early second trimester scan, all births.

Severe SGA ^a^					
Fetal growth ^b^ (centile)	N 69 550	n (%) 1 447	Odds Ratio (95% Confidence Interval)		
			Crude	Adjusted ^c^	Restricted ^d^
>90^th^	7 082	113 (1.6)	0.75 (0.62–0.92)	0.74 (0.60–0.90)	0.73 (0.59–0.89)
90^th^ to 10^th^	55 518	1 167 (2.1)	Ref	Ref	Ref
10^th^ to 2.5^th^	5 407	115 (2.1)	1.01 (0.83–1.23)	1.09 (0.90–1.34)	1.11 (0.90–1.36)
< 2.5^th^	1 543	52 (3.4)	1.62 (1.22–2.15)	1.79 (1.34–2.40)	1.76 (1.30–2.38)

^a^ Birth weight for gestational age below 3^rd^ percentile according the sex specific national reference curve for fetal growth.

^b^ Fetal growth between observed and estimated gestational age at second trimester ultrasound.

^c^ Adjusted for maternal age, BMI, height, parity, smoking, IVF, pre-pregnancy hypertension and pre-pregnancy diabetes mellitus.

^d^ In this model all cases with preeclampsia, gestational hypertension and gestational diabetes mellitus were excluded.

Similar associations were seen between fetal growths below the 2. 5^th^ centile at early second trimester scan and preterm severe SGA (< 37 weeks) and severe SGA in term pregnancies (≥ 37 weeks) (Tables 3 and 4, respectively).

**Table 3:** Risk for small for gestational age (SGA) and birth before 37 weeks of gestation, in relation to the discrepancy between observed and estimated fetal size at early second trimester scan.

Preterm severe SGA ^a^					
Discrepancy ^b^(days)	N 69 550	n (%) 248	Odds Ratio (95% Confidence Interval)		
			Crude	Adjusted model ^c^	Restricted ^d^
>90^th^	14 177	24 (0.3)	0.75 (0.62–0.92)	0.74 (0.60–0.90)	0.73 (0.59–0.89)
10^th^– 90^th^	32 770	199 (0.3)	Ref	Ref	Ref
5^th^- 10^th^	20 508	17 (0.3)	1.01 (0.83–1.23)	1.10 (0.90–1.34)	1.11 (0.90–1.35)
< 5^th^	1 543	8 (0.5)	1.62 (1.22–2.15)	1.80 (1.35–2.41)	1.76 (1.30–2.38)

^a^ Birth weight for gestational age below 3^rd^ percentile according the sex specific national reference curve for fetal growth.

^b^ Fetal growth between observed and estimated gestational age at second trimester ultrasound.

^c^ Adjusted for maternal age, BMI, height, parity, smoking, IVF, pre-pregnancy hypertension and pre-pregnancy diabetes mellitus.

^d^ In this model all cases with preeclampsia, gestational hypertension and gestational diabetes mellitus were excluded.

**Table 4:** Risk for term severe small for gestational age (SGA) infant, defined as a SGA infant born 37 weeks of gestation or later, in relation to the fetal growth at early second trimester scan. Only term pregnancies, total 66 789 pregnancies.

Term SGA ^a^					
Fetal growth ^b^ (centile)	N66 731	n (%) 1 188	Odds Ratio (95% Confidence Interval)		
			Crude	Adjusted ^c^	Restricted ^d^
> 90	6 607	89 (1.3)	0.73 (0.59–0.91)	0.70 (0.56–0.88)	0.71 (0.56–0.89)
10 to 90	53 317	959 (1.8)	Ref	Ref	Ref
<2.5 to >10	5 227	96 (1.8)	1.02 (0.83–1.26)	1.12 (0.90–1.39)	1.12 (0.89–1.39)
≤ 2.5	1 491	44 (2.9)	1.66 (1.22–2.26)	1.87 (1.37–2.56)	1.84 (1.33–2.53)

^a^ Birth weight for gestational age below 3^rd^ percentile according the sex specific national reference curve for fetal growth.

^b^ Fetal growth between observed and estimated gestational age at second trimester ultrasound.

^c^ Adjusted for maternal age, BMI, height, parity, smoking, IVF, pre-pregnancy hypertension and pre-pregnancy diabetes mellitus.

^d^ In this model all cases with preeclampsia, gestational hypertension and gestational diabetes mellitus were excluded.

In the analyses stratified by maternal age, the adjusted ORs for severe SGA were similar in both groups; maternal age < 35 years (aOR 1.77; 95% CI 1.18–2.67) and maternal age 35 years and older (aOR 1.82; 95% CI 1.19–2.75). (Table 5).

**Table 5:** Risk for severe small for gestational age (SGA) in relation to the discrepancy between observed and estimated fetal size at early second trimester scan, all births. Analyses stratified for maternal age.

Maternal age < 35 y			Maternal age ≥ 35 y	
Odds Ratio (95% Confidence Interval)^a^				
	Crude	Adjusted ^c^	Crude	Adjusted^c^
> 90^th^	0.64 (0.51–0.88)	0.66 (0.49–0.87)	0.87 (0.65–1.15)	0.83 (0.63–1.11)
10^th^–90^th^	Ref	Ref	Ref	Ref
2.5^th^–10^th^	0.94 (0.71–1.23)	1.05(0.79–1.39)	1.11 (0.84–1.45)	1.15 (0.86–1.53)
< 2.5^th^	1.50 (1.01–2.25)	1.77 (1.18–2.67)	1.77 (1.19–2.62)	1.82 (1.19–2.75)

^a^ Odds ratio for birth weight for gestational age below 3^rd^ percentile according the sex specific national reference curve for fetal growth.

^b^ Fetal growth between observed and estimated gestational age at second trimester ultrasound.

^c^ Adjusted for maternal age, BMI, height, parity, smoking, IVF, pre-pregnancy hypertension and pre-pregnancy diabetes mellitus.

## Discussion

### Main findings of the study

In this large cohort study, we found that the risk of severe SGA at birth increased with slow fetal growth between a first trimester and an early second trimester scan. The risks of preterm (before 37 weeks) and term (37 weeks or later) severe SGA were at similar levels and highest for fetuses with fetal growth below 2.5^th^ centile at an early second trimester scan.

The main strengths of this study are the prospectively recorded information and the large cohort size, which makes it possible to predict the risk for rare outcomes such as preterm severe SGA. The study is population based, antenatal and obstetric care is free of charge in Sweden, and standardized ultrasound examinations in early pregnancy are commonly performed in the region. Because ultrasound measurements were collected from a large number of units, there might by a variation of performance and interpretation of ultrasound examination. However, all included ultrasound units follow the professional standards for equipment specifications and training, set by Swedish technology assessment in health [26] and by the Swedish Society of Obstetrics and Gynecology. [24] As a large fraction of women did not perform first trimester scans, a potential selection bias cannot be excluded. However, we adjusted for maternal age in the logistic regression analysis. Furthermore, when we stratified our data on maternal age below or 35 years or older, the estimates were generally of similar magnitude for the age groups.

### Interpretation

The terminology for classifying fetuses and newborns who have failed to achieve normal weight is inconsistent. In Sweden, SGA is defined as birth weight of two standard deviations or less the average weight for gestational age and sex.[25] However, internationally SGA is often described as newborns whose birth weight is less than the 10^th^ percentile for gestational age. [27] Since we wanted to focus on intrauterine growth restriction, we defined severe SGA infants as those with birth weight was less than 3^rd^ percentile. Severe SGA might have several causes such as structural or chromosomal abnormalities that affect fetal growth, be constitutionally small or exposed to fetal malnutrition. [10, 11] We excluded infants with malformations and adjusted the analysis for maternal stature and BMI. We included maternal history of diabetes as a covariate in the analysis, because insulin and insulin-like growth factors (IGFs) are implicated in the receptor-mediated regulation of placental growth and transport, trophoblast invasion and placental angiogenesis and may enhance placental and fetal growth. [28, 29] We believe that, after adjusting for possible covariates and by having a narrow definition of SGA (less than the 3^rd^ percentile), we were better able to study fetuses that were truly growth restricted.

Fetuses with IUGR do not achieve their growth potential because of environmental factors that influence placental function. [10] Hypertensive diseases and IUGR are both related to placental insufficiency, caused by an abnormal trophoblast invasion of the uterine spiral arteries that starts at 8–10 weeks and is almost completed by 16–18 weeks of gestation.[30] In our study cohort, rates of hypertensive diseases increased from 3.7% in pregnancies with normal weight infants to 8.9% in those with SGA birth. However, in pregnancies without clinical sings of maternal hypertensive diseases, a fetal growth below the 2.5 ^th^ centile at an early second trimesters was an independent risk factor for severe SGA. Our findings support the hypothesis that IUGR and preeclampsia, that are presumed to share the same etiology, might be two different entities and IUGR can be present without clinical signs of gestational hypertension or preeclampsia. [20, 21, 31–33]

In previous studies, first trimester predictions of severe term SGA have been less strong than predictions of early SGA. [34] Explanations could be differences in study design or that pathological placental development starts later in pregnancy. [34, 35] We observed that slow BPD growth in early pregnancy was associated with an increased risk of term SGA, indicating that early placental dysfunction may be the origin of fetal growth restriction also in term pregnancies.

The increased risk of adverse neonatal outcome in fetuses smaller than expected at early second trimester ultrasound scan has previously been based on comparison of early fetal size in relation to LMP, [14, 15, 36–38] date of embryo transfer (ET) when IVF was used, [5] or by comparing different measurements of fetal biophysical characteristics. [20] By investigating the growth of one biophysical characteristic, BPD, at two occasions, using the same dating formula, we were able to avoid uncertainty introduced by comparing different methods. Repeated ultrasonic scans is the preferred method to investigate fetal growth, and previous research indicates that slow BPD growth rate (below 2.5 percentiles) is a risk factor of IUGR. [17] Fetal size is usually presented and recorded as gestational age in days at routinely ultrasound scan in early pregnancy. Still, in order to make our results accessible to wider clinical and research community and comparable with international research results, we used Z score to express fetal growth.

In summary, growth discrepancy in early pregnancy is indicative of a subgroup of slow-growing fetuses that are at increased risk of severe SGA. Our method, based on two sequential ultrasound measurements of the BPD using the same dating formula, should be more reliable than previously described methods.[5, 20, 21] Our findings are of importance because early recognition of fetuses at risk can improve the neonatal prognosis. [12, 13] Our results indicate that slow early fetal growth is a sign of placental disease, leading to preterm severe SGA as well as to term severe SGA. Today, most pregnant women in high income countries undergo ultrasound examinations in first and second trimester. However, before proper clinical evaluation, we cannot justify the recommendation for routinely use of the method.

## Conclusion

Fetal growth below the 2.5 ^th^ centile during early pregnancy increases the risk of preterm and term severe SGA birth, also in pregnancies without hypertensive diseases. These findings may be used to identify pregnancies at risk and thereby optimize prenatal management.

## Supporting information

S1 Table Risk for intermediate small for gestational age (SGA) in relation to the discrepancy between observed and estimated fetal size at early second trimester scan, all births. (DOCX)
