# Supplementary material for: Slow fetal growth between first and early second trimester ultrasound scans and risk of small for gestational age (SGA) birth
Source: PLoS One. 2017 Sep 21;12(9):e0184853. doi: 10.1371/journal.pone.0184853 (PMC5608242; doi:10.1371/journal.pone.0184853)
Supplement: S1 Table — (DOCX) [file pone.0184853.s001.docx]

**Supplementary Table 1**

**Risk for intermediate small for gestational age (SGA) in relation to the discrepancy between**

**observed and estimated fetal size at early second trimester scan, all births**.

| **Intermediate SGA ^a^** | | | | | | |
| --- | --- | --- | --- | --- | --- | --- |
| **Fetal growth ^b^**  **(centile)** | N  69 550 | n (%)  4 502 | Odds Ratio (95% Confidence Interval) | | | |
|  |  |  | Crude | | Adjusted ^c^ | Restricted ^d^ |
| >90 | 1 543 | 426 (6.0) | | 0.92 (0.83-1.02) | 0.90 (0.80-1.00) | 0.89 (0.80-0.99) |
| 10-90 | 55 518 | 3 599 (6.5) | | Ref | Ref | Ref |
| 2.5-10 | 5 407 | 368 (6.5) | | 1.05 (0.94-1.17) | 1.10 (0.98-1.24) | 1.10 (0.98-1.24) |
| < 2.5 | 1 542 | 109 (7.1) | | 1.09 (0.90-1.33) | 1.16 (0.94-1.42) | 1.18 (0.96-1.45) |

^a^ Birth weight for gestational age between 3^rd^ and 10^th^ percentile according the sex specific national reference curve for fetal growth.

^b^ Fetal growth between observed and estimated gestational age at second trimester ultrasound.

^c^ Adjusted for maternal age, BMI, height, parity, smoking, IVF, pre-pregnancy hypertension and pre-pregnancy diabetes mellitus.

^d^ In this model all cases with preeclampsia, gestational hypertension and gestational diabetes mellitus were excluded.
